# Analysis of acute pancreatitis associated with SGLT-2 inhibitors and predictive factors of the death risk: Based on food and drug administration adverse event report system database

**DOI:** 10.3389/fphar.2022.977582

**Published:** 2022-11-18

**Authors:** Lin Zhang, Wei Mao, Xingxing Li, Xiaowen Wang, Jifang Liu, Sang Hu, Jing Hu

**Affiliations:** ^1^ Department of Pharmacy, Southwest Hospital of Army Medical University (Third Military Medical University), Chongqing, China; ^2^ Department of Pharmacy, Nanan People’s Hospital of Chongqing, Chongqing, China

**Keywords:** SGLT-2i, acute pancreatitis, FAERS, adverse events, risk profile

## Abstract

**Background and objectives:** The US FDA and Health Canada have successively published potential red flags for acute pancreatitis caused by sodium-dependent glucose transporter 2 inhibitors (SGLT-2i). However, existing studies have focused on case reports. We aimed to assess the possible association of SGLT-2i with acute pancreatitis by analyzing postmarketing adverse events reported in the FDA adverse event reporting system (FAERS), to explore risk factors for SGLT-2i-related acute pancreatitis death, and to build a nomogram.

**Methods and Results:** We used a disproportionality analysis of suspected acute pancreatitis-related reports in the FAERS database of patients from the use of SGLT-2i from the first quarter of 2013 to the fourth quarter of 2021. Single-factor and multi-factor logistic regression analyses were performed using the relevant clinical information of patients, and risk factors were combined with the age of patients to construct a SGLT-2i risk prediction model for acute pancreatitis-related death. A total of 757 reports were retrieved. The largest number of acute pancreatitis-related cases were caused by canagliflozin (317 reports), which was also the strongest agent associated with acute pancreatitis, with the information component (IC 2.41, lower 95% one-sided confidence interval 2.16), the reporting odds ratio (ROR 5.37, 95% two-sided confidence interval 4.8–5.99), and the empirical Bayesian geometric mean (EBGM 5.32, lower 90% one-sided confidence interval 4.85). The median time to acute pancreatitis was 54 (interquartile range [IQR] 14–131) days, and approximately 83% of adverse events occurred within 6 months. Odds ratio(OR) adjusted by acute pancreatitis and the coadministration of SGLT-2i with dipeptidyl peptidase 4 inhibitor (DPP-4i), glucagon-like peptide 1 analog (GLP-1RA), and angiotensin converting enzyme inhibitor (ACEIs) was 1.39, 1.97, and 1.34, respectively, all of which were statistically significant. Logistic regression analysis showed that different SGLT-2i type and their combinations with statins were independent risk factors for acute pancreatitis mortality in the patients (*p* < 0.05). The mortality risk prediction model showed good discrimination and clinical applicability in both the training set (AUC 0.708) and the validation set (AUC 0.732).

**Conclusion:** SGLT-2i may increase the risk of acute pancreatitis especially within the first 6 months of drug administration. Combination with DPP-4i, GLP-1RA or ACEIs significantly increases the risk of acute pancreatitis. In addition, different SGLT-2i type and their combination with statins are risk factors that can predict the risk of death following acute pancreatitis.

## Introduction

Sodium-dependent glucose co-transporter 2 inhibitors (SGLT-2i) are a novel class of oral hypoglycemic agents, which reduce blood glucose concentrations by inhibiting the sodium-dependent glucose co-transporter 2, thereby reducing the reabsorption of glucose by the kidney, and increasing the excretion of glucose in the urine (Kramer and Zinman, 2019). SGLT-2i activity is independent of the glucose-dependent insulin pathway. It can reduce the risk of hypoglycemia and has a hypoglycemic effect. A large number of clinical studies have confirmed that treatment with SGLT-2i can effectively reduce the mortality from cardiovascular diseases and the hospitalization rate from heart failure (Kosiborod et al., 2018; Palmer et al., 2021), and delays the deterioration of renal function (Sarafidis et al., 2021; Sen and Heerspink, 2021), and thus, has broad prospects for clinical application.

With the wide use of these drugs, their safety has attracted increased attention. Some case reports have indicated that SGLT-2i leads to acute pancreatitis (Chowdhary et al., 2015; Verma, 2016; McIntire and Bayne, 2018; Sujanani et al., 2020; Zeidan Jr et al., 2020). In 2016, the United States Food and Drug Administration (US FDA) released a potential hazard alert of SGLT-2i for acute pancreatitis (US Food and Drug Administration, 2016). In 2018, the Ministry of Health of Canada reported the results of SGLT-2i risk assessment for pancreatitis caused by hypoglycemic drugs, and suggested that SGLT-2i might be related to acute pancreatitis (Health Canada, 2022). Acute pancreatitis is an acute, inflammatory, potentially life-threatening condition of the pancreas and is the leading gastrointestinal cause of hospitalization. There are numerous established etiologies of acute pancreatitis, among which gallstones and alcohol are the most common (40%–70% and 25%–35%, respectively); drugs are a relatively rare cause of acute pancreatitis, accounting for about 2% of cases (Weissman et al., 2020). Most cases of drug-induced pancreatitis are mild to moderate in severity, but some cases may still lead to serious complications and even death (Wolfe et al., 2020). Important adverse reactions described in the SGLT-2i package insert include genital fungal infection, diabetic ketoacidosis, perineal necrotizing fasciitis, amputation; however, acute pancreatitis has not yet been included, which may be related to the lack of a large number of clinical trials. In the absence of a randomized controlled trial of an appropriate size, physicians and patients should, whenever possible, look elsewhere for evidence to assess the risk of SGLT-2i-associated acute pancreatitis. Real-world-based pharmacovigilance may be a suitable method of finding such evidence.

The FDA Adverse Event Reporting System (FAERS) is a repository of spontaneously reported adverse drug events that support the postmarketing drug safety surveillance of the FDA (Rodriguez et al., 2001) and is the largest real-world pharmacovigilance program. The aim of this study was to collect, screen, and statistically analyze relevant data in the FAERS database, and to conduct a signal mining analysis of suspected ADRs generated by SGLT-2i, focusing on the potential correlation between SGLT-2i and acute pancreatitis. The study also explored risk factors for acute pancreatitis due to SGLT-2i in order to construct a risk model for predicting acute pancreatitis mortality to ensure its safe and rational clinical application.

## Materials and methods

### Data source and collection

Reports of acute pancreatitis using SGLT-2i (canagliflozin, dapagliflozin, empagliflozin, ertugliflozin) from the first quarter 2013 to the fourth quarter 2021 in the FAERS database were collected and adverse events were coded by preferred terms (PTs) according to the Medical Dictionary for Regulatory Activities Terminology (MedDRA). Acute pancreatitis included: necrotizing pancreatitis, acute pancreatitis, pancreatitis, pancreatic infection, hemorrhagic necrotic pancreatitis, hemorrhagic pancreatitis, ischemic pancreatitis, pancreatic abscess, pancreatic phlegmon. We collected clinical information relevant to patients with acute pancreatitis (sex, age, body weight, reporting area, reporting date, reporting source, drug combinations, concomitant disease, and serious adverse events), removed duplicate records, and ruled out the absence of sex and age dependence.

### Data mining

For each drug-event pair IC, ROR and EBGM were calculated to detect drug-event pairs with higher-than-expected reporting rates versus all other drugs in the FDA registry. The intersection of the three algorithms indicated valid the adverse drug events (ADE) (Table 1).

**TABLE 1:** Three algorithms for signal detection.

Algorithms	Equation	Criteria
BCPNN	IC=log2a(a+b+c+d)(a+b)(a+c)	$IC_{025}>0$
	$IC_{025}=e^{\ln\left(IC\right)-1.96\sqrt{\left(1/a+1/b+1/c+1/d\right)}}$	
ROR	ROR=a/c/b/d	$a\geq 3,ROR_{025}>1$
	$ROR_{025}=e^{\ln\left(ROR\right)-1.96\sqrt{\left(1/a+1/b+1/c+1/d\right)}}$	
MGPS	EBGM=a(a+b+c+d)/(a+c)/(a+b)	${EBGM}_{05}>1$
	${EBGM}_{05}=e^{\ln\left(EBGM\right)-1.641.96\sqrt{\left(1/a+1/b+1/c+1/d\right)}}$	

where, *a* is the number of reports containing both the target drug and the target adverse event; *b* is the number of reports containing the target drug with other adverse events; *c* is the number of reports containing the target adverse event with other drugs; *d* is the number of reports containing other drugs and other adverse events; IC_025_, lower limit of the 95% CI of the IC; ROR_025_, lower limit of the 95% CI of the ROR; EBGM_05_, lower limit of 90% CI of the EBGM.

### Statistical analysis

We use descriptive statistics to summarize the clinical characteristics of the cases. The Chi-square test was used to compare the category variables between groups, and the Wilcoxon test was used to determine the statistical significance of continuous variables. A *p*-value of <0.05 was considered statistically significant. A model for predicting the risk of death caused by SGLT-2i in acute pancreatitis was developed. According to the discriminant ability of the area under curve (AUC) value, the AUC values varied from 0.5 to 1.0, where 0.5 represented random chance and 1.0 represented the perfect fit. AUC values greater than 0.7 suggested a reasonable estimation. The calibration curve was used to evaluate the consistency between the prediction probability and the actual occurrence. The clinical practicability was judged by the decision curve. MySQL database (version 8.0.28) and R software (version 4.1.3) were used for data operation and statistical analysis.

## Results

### Adverse drug event reports basic information

A total of 76,872 acute pancreatitis related adverse events were recorded from the first quarter of 2013 to the fourth quarter of 2021, 757 of which were related to SGLT-2i, including 317 for canagliflozin, 150 for dapagliflozin, 287 for empagliflozin, and three for ertugliflozin. The incidence of acute pancreatitis in men (56.7%) was higher than that in women (43.3%). The average age was 56.2 years, of which, ages 18–64 years accounted for 75%. The mean body weight was 95.8 kg (data available in the 334/757 report). Major reports of acute pancreatitis after 2015 were reported by medical staff, and accounted for 67.2% of cases, mainly occurring in the United States, Canada, and the United Kingdom. Serious adverse events accounted for 99.3% of reports, with 70.0% describing inpatient treatment or prolonged hospitalization and 4.2% cases of patient deaths. The basic characteristics of adverse events are described in Table 2.

**TABLE 2:** Demographic and clinical characteristics of patients with acute pancreatitis.

Characteristics	Canagliflozin (N = 317)	Dapagliflozin (N = 150)	Empagliflozin (N = 287)	Ertugliflozin (N = 3)	SGLT-2i (N = 757)	*p*-value
Gender						0.966
Male	177 (55.8%)	86 (57.3%)	164 (57.1%)	2 (66.7%)	429 (56.7%)	
Female	140 (44.2%)	64 (42.7%)	123 (42.9%)	1 (33.3%)	328 (43.3%)	
Age						0.088
Mean (SD)	55.2 (11.8)	56.4 (10.6)	57.1 (13.0)	63.3 (11.5)	56.2 (12.1)	
Median (IQR)	55 (48, 63)	56.3 (50, 64)	59 (50, 66)	70 (60, 70)	57 (49, 64)	
<18	0 (0%)	0 (0%)	1 (0.3%)	0 (0%)	1 (0.1%)	
18–64	248 (78.2%)	113 (75.3%)	206 (71.8%)	1 (33.3%)	568 (75.0%)	
65–80	68 (21.5%)	36 (24.0%)	70 (24.4%)	2 (66.7%)	176 (23.2%)	
>80	1 (0.3%)	1 (0.7%)	10 (3.5%)	0 (0%)	12 (1.6%)	
Weight						0.812
Mean (SD)	97.5 (26.7)	95.2 (25.0)	94.0 (20.1)	84.0 (NA)	95.8 (24.4)	
Median (IQR)	94.2 (81.7, 110)	90 (76, 107.4)	94.8 (76.6, 108)	84 (84, 84)	93 (77, 108.3)	
Unknown	172 (54.3%)	65 (43.3%)	184 (64.1%)	2 (66.7%)	423 (55.9%)	
Reporting year						<0.001
2013	2 (0.6%)	0 (0%)	0 (0%)	0 (0%)	2 (0.3%)	
2014	26 (8.2%)	2 (1.3%)	0 (0%)	0 (0%)	28 (3.7%)	
2015	82 (25.9%)	16 (10.7%)	13 (4.5%)	0 (0%)	111 (14.7%)	
2016	82 (25.9%)	24 (16.0%)	18 (6.3%)	0 (0%)	124 (16.4%)	
2017	60 (18.9%)	18 (12.0%)	28 (9.8%)	0 (0%)	106 (14.0%)	
2018	33 (10.4%)	21 (14.0%)	56 (19.5%)	0 (0%)	110 (14.5%)	
2019	12 (3.8%)	25 (16.7%)	67 (23.3%)	2 (66.7%)	106 (14.0%)	
2020	12 (3.8%)	26 (17.3%)	43 (15.0%)	1 (33.3%)	82 (10.8%)	
2021	8 (2.5%)	18 (12.0%)	62 (21.6%)	0 (0%)	88 (11.6%)	
Notifier type						0.319
Physician	119 (37.5%)	56 (37.3%)	132 (46.0%)	1 (33.3%)	308 (40.7%)	
Pharmacist	36 (11.4%)	12 (8.0%)	35 (12.2%)	0 (0%)	83 (11.0%)	
Other health professional	46 (14.5%)	21 (14.0%)	49 (17.1%)	1 (33.3%)	117 (15.5%)	
Lawyer	0 (0%)	1 (0.7%)	2 (0.7%)	0 (0%)	3 (0.4%)	
Consumer or non-health professional	106 (33.4%)	33 (22.0%)	65 (22.6%)	1 (33.3%)	205 (27.1%)	
Unknown	10 (3.2%)	27 (18.0%)	4 (1.4%)	0 (0%)	41 (5.4%)	
Country						<0.001
America	255 (80.4%)	91 (60.7%)	152 (53.0%)	1 (33.3%)	499 (65.9%)	
Canada	15 (4.7%)	0 (0%)	32 (11.1%)	0 (0%)	47 (6.2%)	
England	2 (0.6%)	21 (14.0%)	12 (4.2%)	0 (0%)	35 (4.6%)	
Japan	3 (0.9%)	0 (0%)	8 (2.8%)	0 (0%)	11 (1.5%)	
Brazil	0 (0%)	8 (5.3%)	6 (2.1%)	0 (0%)	14 (1.8%)	
Germany	0 (0%)	1 (0.7%)	17 (5.9%)	0 (0%)	18 (2.4%)	
Spain	0 (0%)	1 (0.7%)	7 (2.4%)	2 (66.7%)	10 (1.3%)	
Other	42 (13.2%)	28 (18.7%)	53 (18.5%)	0 (0%)	123 (16.2%)	
Serious	316 (99.7%)	146 (97.3%)	287 (100%)	3 (100%)	752 (99.3%)	0.008
Death	16 (5.0%)	3 (2.0%)	11 (3.8%)	2 (66.7%)	32 (4.2%)	<0.001
Hospitalization	238 (75.1%)	99 (66.0%)	190 (66.2%)	3 (100%)	530 (70.0%)	0.04
Disabling	8 (2.5%)	7 (4.7%)	5 (1.7%)	0 (0%)	20 (2.6%)	0.335
Life threatening	35 (11.0%)	8 (5.3%)	38 (13.2%)	0 (0%)	81 (10.7%)	0.077

### Adverse drug events associated with different SGLT-2i

Treatment with SGLT-2i was significantly associated with acute pancreatitis, with IC (2.31, IC_025_ 2.15), ROR (5.03, 95% CI 4.68–5.41), and EBGM (4.97, EBGM_05_ 4.68). Canagliflozin had the strongest potential associations with acute pancreatitis occurrence, with IC (2.41, IC_025_ 2.16), ROR (5.37, 95% CI 4.8–5.99), and EBGM (5.32, EBGM_05_ 4.85). The results are presented in Table 3.

**TABLE 3:** Association between different SGLT2 inhibitors and acute pancreatitis occurrence.

Drug	A	IC(IC_025_)	ROR (95% CI)	EBGM(EBGM_05_)
SGLT-2i	757	2.31 (2.15)	5.03 (4.68, 5.41)	4.97 (4.68)
Canagliflozin	317	2.41 (2.16)	5.37 (4.8, 5.99)	5.32 (4.85)
Dapagliflozin	150	2.26 (1.92)	4.8 (4.09, 5.64)	4.77 (4.17)
Empagliflozin	287	2.25 (2)	4.78 (4.25, 5.37)	4.74 (4.31)
Ertugliflozin	3	1.83 (0.59)	3.58 (1.15, 11.12)	3.57 (1.38)

### Time interval between SGLT-2i initiation and acute pancreatitis

After excluding the reports of missing occurrence time, a total of 180 reports were finally included. The median time to onset of acute pancreatitis due to SGLT-2i was 54 (IQR 14–131)days, with approximately 83% of reported cases occurred within the first 6 months after drug initiation. The results are presented in Supplementary Figure S1.

### Analysis of the influence of medication combinations on acute pancreatitis caused by SGLT-2i

**FIGURE 1:**
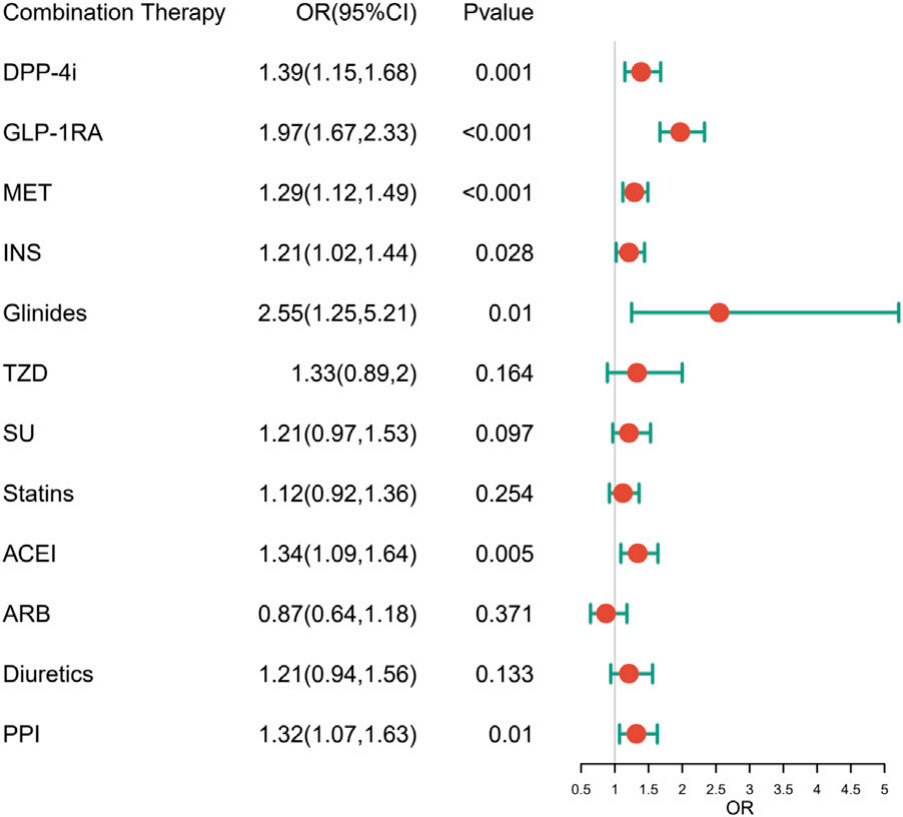
The risk of acute pancreatitis induced by combination treatments.

After adjustment for potential confounders, the risk of acute pancreatitis of SGLT-2i combined with DPP-4i, GLP-1RA, metformin, insulin, glinide, ACEIs and PPIs was higher than that of SGLT-2i monotherapy (adjusted OR 1.39, 1.97, 1.29, 1.21, 2.55, 1.34, 1.32, respectively). The results are presented in Figure 1.

### Nomogram construction and validation

After excluding reports with missing sex, age, country, and simultaneous use of two or more SGLT-2i data, 711 reports describing acute pancreatitis following the use of SGLT-2i were extracted. The reported were randomly divided 1:1 into a training set and validation set (Supplementary Table S1).

**FIGURE 2:**
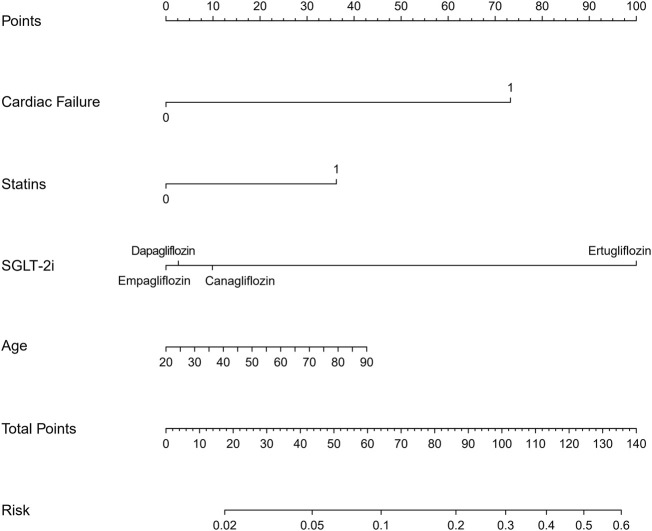
Nomogram model for predicting death in patients with acute pancreatitis.

**FIGURE 3:**
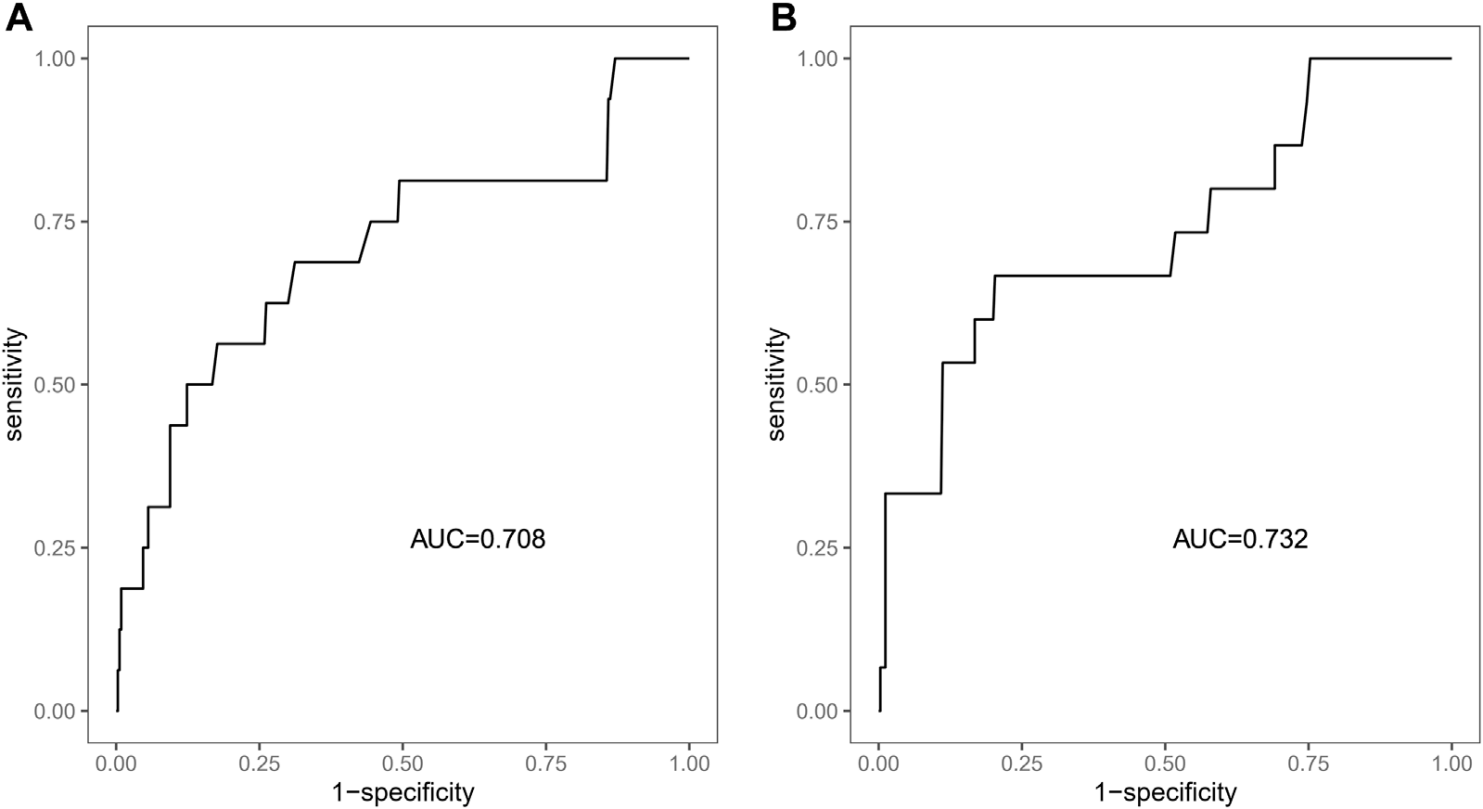
ROC curve of the nomogram to predict the risk of death. **(A)** ROC curve of the training set, **(B)** ROC curve of the validation set.

**FIGURE 4:**
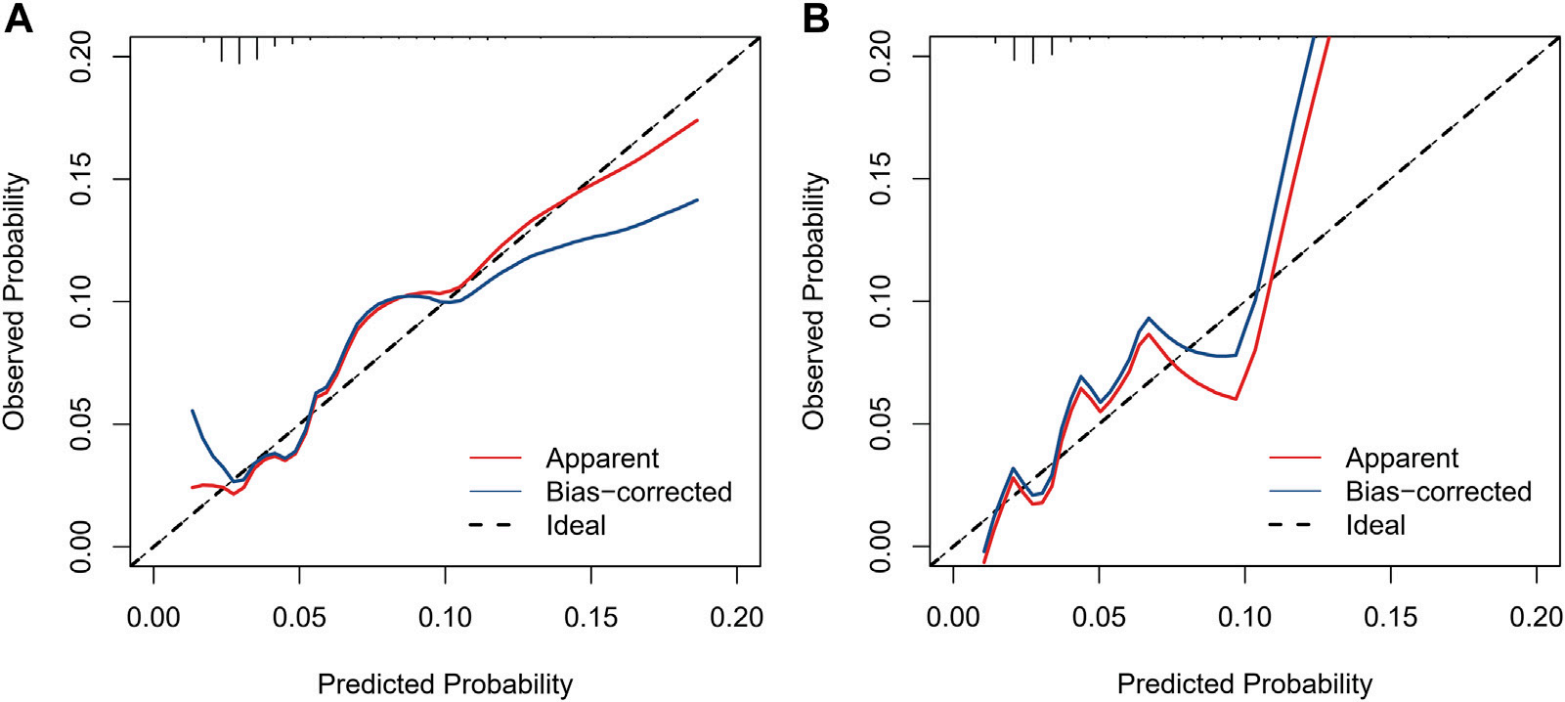
Calibration curves of the nomogram to predict the risk of death. **(A)** Calibration curve of the training set, **(B)** calibration curve of the validation set.

**FIGURE 5:**
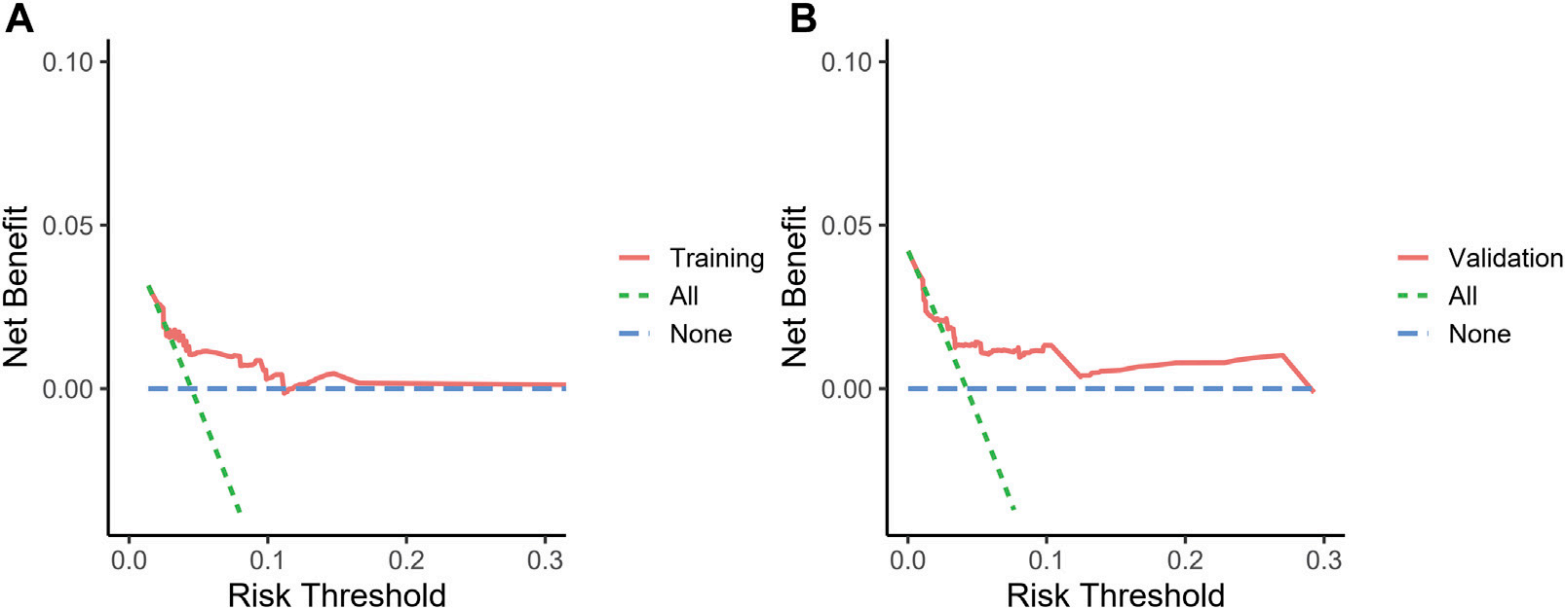
Decision curve the nomogram to predict the risk of death. **(A)** Decision curve of the training set, **(B)** decision curve of the validation set.

We included sex, age, country, SGLT-2i type, concomitant medications, and concomitant diseases into the logistic regression model. The single factor logistic regression showed that the combination of statins, concomitant heart failure, and SGLT-2i type were the risk factors for death in patients with acute pancreatitis. Multivariate logistic regression analysis revealed that the combination of statins and the different SGLT-2i were independent risk factors for acute pancreatitis death (*p* < 0.05), (Supplementary Table S2). Risk factors in the logistic regression model and the age were used to construct a nomogram model (Figure 2). The nomogram was validated in the training set and the validation set. The resulting ROC curve analysis showed that the AUC of the training set and that of the validation set were 0.708 and 0.732, respectively, showing good model discrimination (Figures 3A,B). A bootstrap self-sampling method was used to verify the model. The results of 2,000 self-sampling and internal verification procedures showed that the Mean absolute error between the predicted risk and actual risk of acute pancreatitis death in the training set was 0.009, and the mean absolute error in the verification set was 0.013, showing good consistency (Figures 4A,B). The decision curve shows a good net gain when the threshold probability is in the range of about 0.02–0.05 (Xiao et al., 2016; Ingraham et al., 2021) (Figures 5A,B).

## Discussion

Our study showed that SGLT-2i might increase the risk of acute pancreatitis, which was consistent with the conclusion that SGLT-2i treatment presented potential hazard for acute pancreatitis, in accordance with the warning released by the US FDA in 2016 (US Food and Drug Administration, 2016). In the present study, we have developed a risk prediction model for acute pancreatitis death for patients treated with SGLT-2i that exhibits good discrimination (training set AUC:0.708, verification set AUC:0.732) and calibration performance with potential clinical value.

Overall, from the first quarter of 2013 to the fourth quarter of 2021, there were 757 reports complete with age and sex data describing SGLT-2i and acute pancreatitis in the FAERS database. The median age of the patients was 57 years, and patients aged 18–64 years accounted for 75% of the patients, which was consistent with the age groups reported by Huilin et al. (Tang et al., 2020). We found that men (56.7%) were more prone to acute pancreatitis than women (43.3%), which might be related to the fact that men were more likely to cause alcoholic acute pancreatitis (Lankisch et al., 2001).

In our study, health care professionals reported 67.2% of acute pancreatitis cases and consumers (including three lawyers) reported 27.5% of cases. Excluding the 2 years since its launch, the number of reported cases have been more than 100 per year since 2015, but the number has declined since 2020 (82 in 2020 and 88 in 2021), which may be related to the global outbreak of SARS-CoV-2 at the end of 2019. Approximately 65.9% of the reports derived from the United States, and may be related to the fact that FAERS was established in the United States. Acute pancreatitis accounts for 70.0% of hospitalized patients or patients with prolonged hospitalization, 4.2% of patients died, 2.6% of patients were disabled, and 10.7% of patients experienced life-threatening reactions. The results of this study reflect that acute pancreatitis is a serious disease requiring special attention.

The largest number of acute pancreatitis-related reports were due to canagliflozin, with 317 in total, accounting for 41.9% of the overall reports. Meanwhile, canagliflozin was also the most acute pancreatitis-related, with IC (2.41, IC_025_ 2.16), ROR (5.37, 95% CI 4.8–5.99), and EBGM (5.32, EBGM_05_ 4.85). Although the reported number of reactions due to empagliflozin was higher than those attributed to Dapagliflozin (287:150), acute pancreatitis-associated events were weaker, with IC (2.25: 2.26), ROR (4.78: 4.8) and EBGM (4.74: 4.77), which might be related to the higher selectivity of empagliflozin (Grempler et al., 2012). Ertugliflozin acute pancreatitis associated events were the weakest, which may be related to its later launch on the market and the limited number of reports (only 3).

We also observed that the median time to onset of SGLT-2i-associated acute pancreatitis was 54 (IQR 14–131) days, with approximately 83% of reported cases occurred within the first 6 months after drug initiation. In the published reports of SGLT-2i caused acute pancreatitis, the time interval from drug initiation to the onset of acute pancreatitis adverse drug reactions ranged from 5–104 days (Chowdhary et al., 2015; Srivali et al., 2015; Verma, 2016; Lightbourne et al., 2017; Patel et al., 2017; Gutch et al., 2018; McIntire and Bayne, 2018; Sujanani et al., 2020; Zeidan Jr et al., 2020). As can be seen, the majority of our results consistent with them, which further indicated that SGLT-2i may increase the risk of acute pancreatitis, especially in the early stage of medication.

To determine whether coadministration with other medications influenced the development of acute pancreatitis, we compared the risk of acute pancreatitis associated with combined drug treatment with that of SGLT-2i monotherapy. The results showed that SGLT-2i combined with DPP-4i, GLP-1RA, metformin, insulin, glinide, ACEI and PPI showed an increased risk of acute pancreatitis (adjusted OR 1.39, 1.97, 1.29, 1.21, 2.55, 1.34, and 1.32, respectively). This is consistent with the increased risk of acute pancreatitis with DPP-4 inhibitors reported by Doni et al. (Doni et al., 2022). It has previously been reported that GLP-1RA may cause acute pancreatitis (Smits and Van Raalte, 2021; AlSaadoun et al., 2022), but two recent network meta-analyses revealed that DPP-4 may increase the risk of acute pancreatitis, while GLP-1RA seems to have no effect (Kanie et al., 2021; Zhuo et al., 2021). However, there are no reports indicating whether the combination of GLP-1RA with SGLT-2i increases this risk. Our study suggests that the combination of ACEIs with SGLT-2i may increase the risk of acute pancreatitis. Acute pancreatitis can be induced by ACEIs itself (Jones et al., 2015; Hussain et al., 2020), and whether the combined use of the two has additive side effects requires additional clinical studies for verification. Previous studies have shown that pantoprazole has anti-inflammatory properties *in vivo* and weakens the course of acute pancreatitis (Hackert et al., 2010). However, subsequent studies have reported that PPI did not affect the clinical outcome of patients with acute pancreatitis (Murata et al., 2015; Ma et al., 2017), and there are separate cases reports describing the rare side effects of PPI (Youssef et al., 2005; Ocal et al., 2014). Therefore, further clinical studies are needed to evaluate the risk of acute pancreatitis caused by SGLT-2i when combined with PPI. Insulin can be used as a treatment option for acute pancreatitis induced by hypertriglyceridemia (Song et al., 2019), and in turn, hypertriglyceridemia is a common cause of acute pancreatitis (Yang and McNabb-Baltar, 2020). The increased risk of acute pancreatitis associated with the use of SGLT-2i in combination with insulin is not known and may be related to the patient’s own disease. Only a single case of acute pancreatitis caused by metformin and glinide has been reported (Alsubaie and Almalki, 2013; Udongwo et al., 2021), and relevant clinical studies are lacking.

In recent years, the mortality rate of acute pancreatitis (approximately 2%–5%) (Xiao et al., 2016; Ingraham et al., 2021) has decreased. Early assessment of the prognosis of patients is crucial to control the progression of the disease. In the present study, we developed a model to predict the risk of acute pancreatitis related death after administration of SGLT-2i based on four predictors: age, type of SGLT-2i, statin use, and concomitant heart failure. The analysis of ROC curve, calibration curve, and decision curve results showed that the model had good discrimation ability, calibration ability, and clinical applicability in both the training set and the validation set. The four predictors we used are readily available in the clinic, and the model allows clinicians to quickly assess the prognosis of such patients using this simple and intuitive predictive tool.

This study also has several limitations. First, FAERS is based on a self-reporting system, with the potential for missing data or reports, repeated reports, and inaccurate reports, which may lead to a bias of research results. Second, the information in the reports has not been verified, and the signals monitored only represent potential associations rather than causality. Third, the lack of information on the population in which SGLT-2i was administered prevented the calculation of the incidence of acute pancreatitis. Moreover, data such as medication dose and treatment duration could not be included our prognosis model. Finally, we constructed a prognostic model that was validated with internal data only and not using external data. Despite these limitations, this study was the first to develop a nomogram based on FAERS data for predicting acute pancreatitis deaths following treatment with SGLT-2i.

## Conclusion

Overall, this study used different statistical methods to analyze the relationship between SGLT-2i and acute pancreatitis and found that SGLT-2i might increase the risk of occurrence of acute pancreatitis. Among these methods, the signal of canagliflozin was significantly higher than that of other SGLT-2i. Drugs such as DPP-4i, GLP-1RA, and ACEIs may increase the risk of acute pancreatitis caused by SGLT-2i. Most reported cases occurred within the first 6 months after drug initiation. In addition, we found that the type of SGLT-2i and the combination with statins may be the risk factors for predicting death in acute pancreatitis. Therefore, we propose that clinical monitoring during clinical treatment should be strengthened, especially in the early stage of drug administration, to vigilance the occurrence of acute pancreatitis.

## Data Availability

The original contributions presented in the study are included in the article/Supplementary Material, further inquiries can be directed to the corresponding author.
