# Supplementary material for: Analysis of acute pancreatitis associated with SGLT-2 inhibitors and predictive factors of the death risk: Based on food and drug administration adverse event report system database
Source: Front Pharmacol. 2022 Nov 18;13:977582. doi: 10.3389/fphar.2022.977582 (PMC9716078; doi:10.3389/fphar.2022.977582)
Supplement: Supplementary file 1 [file DataSheet1.ZIP › Supplementary Table/Supplementary Table S1.docx]

Supplementary Table S1. Clinicopathological characteristics of all patients

| **Characteristics** | **Training** | **Validation** | **Overall** | **P value** |
| --- | --- | --- | --- | --- |
|  | **(N=356)** | **(N=355)** | **(N=711)** |  |
| **Sex** |  |  |  | 0.088 |
| Male | 190 (53.4%) | 213 (60.0%) | 403 (56.7%) |  |
| Female | 166 (46.6%) | 142 (40.0%) | 308 (43.3%) |  |
| **Age** |  |  |  | 0.994 |
| Mean (SD) | 56.3 (12.2) | 56.1 (12.3) | 56.2 (12.2) |  |
| Median (IQR) | 57 (49.8, 65) | 57 (48.5, 64) | 57 (49, 65) |  |
| **SGLT-2i** |  |  |  | 0.846 |
| Canagliflozin | 146 (41.0%) | 140 (39.4%) | 286 (40.2%) |  |
| Dapagliflozin | 66 (18.5%) | 73 (20.6%) | 139 (19.6%) |  |
| Empagliflozin | 142 (39.9%) | 141 (39.7%) | 283 (39.8%) |  |
| Ertugliflozin | 2 (0.6%) | 1 (0.3%) | 3 (0.4%) |  |
| **DPP-4i** | 64 (18.0%) | 56 (15.8%) | 120 (16.9%) | 0.494 |
| **GLP-1RA** | 88 (24.7%) | 91 (25.6%) | 179 (25.2%) | 0.846 |
| **MET** | 175 (49.2%) | 175 (49.3%) | 350 (49.2%) | 1 |
| **INS** | 80 (22.5%) | 82 (23.1%) | 162 (22.8%) | 0.913 |
| **Glinides** | 4 (1.1%) | 4 (1.1%) | 8 (1.1%) | 1 |
| **TZD** | 9 (2.5%) | 12 (3.4%) | 21 (3.0%) | 0.653 |
| **SU** | 37 (10.4%) | 39 (11.0%) | 76 (10.7%) | 0.893 |
| **Statins** | 53 (14.9%) | 58 (16.3%) | 111 (15.6%) | 0.668 |
| **ACEI** | 48 (13.5%) | 53 (14.9%) | 101 (14.2%) | 0.656 |
| **ARB** | 23 (6.5%) | 20 (5.6%) | 43 (6.0%) | 0.76 |
| **Diuretics** | 29 (8.1%) | 30 (8.5%) | 59 (8.3%) | 0.991 |
| **PPI** | 44 (12.4%) | 51 (14.4%) | 95 (13.4%) | 0.499 |
| **Cardiac Failure** | 2 (0.6%) | 3 (0.8%) | 5 (0.7%) | 0.997 |
| **Cholesterol Increased** | 10 (2.8%) | 8 (2.3%) | 18 (2.5%) | 0.816 |
| **Coronary Disease** | 1 (0.3%) | 3 (0.8%) | 4 (0.6%) | 0.614 |
| **Gastrooesophageal Reflux Disease** | 7 (2.0%) | 9 (2.5%) | 16 (2.3%) | 0.796 |
| **Hypertension** | 23 (6.5%) | 28 (7.9%) | 51 (7.2%) | 0.554 |
| **Triglycerides Increased** | 5 (1.4%) | 2 (0.6%) | 7 (1.0%) | 0.45 |
| **Country** |  |  |  | 0.046 |
| CA | 31 (8.7%) | 16 (4.5%) | 47 (6.6%) |  |
| US | 250 (70.2%) | 249 (70.1%) | 499 (70.2%) |  |
| Other | 75 (21.1%) | 90 (25.4%) | 165 (23.2%) |  |
| **Death** | 16 (4.5%) | 15 (4.2%) | 31 (4.4%) | 1 |
