# Supplementary material for: Analysis of acute pancreatitis associated with SGLT-2 inhibitors and predictive factors of the death risk: Based on food and drug administration adverse event report system database
Source: Front Pharmacol. 2022 Nov 18;13:977582. doi: 10.3389/fphar.2022.977582 (PMC9716078; doi:10.3389/fphar.2022.977582)
Supplement: Supplementary file 1 [file DataSheet1.ZIP › Supplementary Table/Supplementary Table S2.docx]

Supplementary Table S2. Logistics regression of univariate and multivariate analyses

| **Characteristics** | **Univariate analysis** | | **Multivariate analysis** | |
| --- | --- | --- | --- | --- |
|  | **OR(95%CI)** | **P value** | **OR(95%CI)** | **P value** |
| Statins | 3.74(1.3,10.77) | 0.015 | 3.81(1.26,11.55) | 0.018 |
| Cardiac Failure | 22.6(1.35,379.04) | 0.03 | 19.97(0.95,418.02) | 0.054 |
| Canagliflozin | Ref |  | Ref |  |
| Dapagliflozin | 0.95(0.24,3.78) | 0.937 | 0.72(0.16,3.23) | 0.668 |
| Empagliflozin | 0.72(0.22,2.34) | 0.59 | 0.72(0.22,2.37) | 0.584 |
| Ertugliflozin | 19.86(1.12,351.61) | 0.042 | 27.99(1.53,511.53) | 0.025 |
