# Supplementary figures and images for: Analysis of acute pancreatitis associated with SGLT-2 inhibitors and predictive factors of the death risk: Based on food and drug administration adverse event report system database
Source: Front Pharmacol. 2022 Nov 18;13:977582. doi: 10.3389/fphar.2022.977582 (PMC9716078; doi:10.3389/fphar.2022.977582)

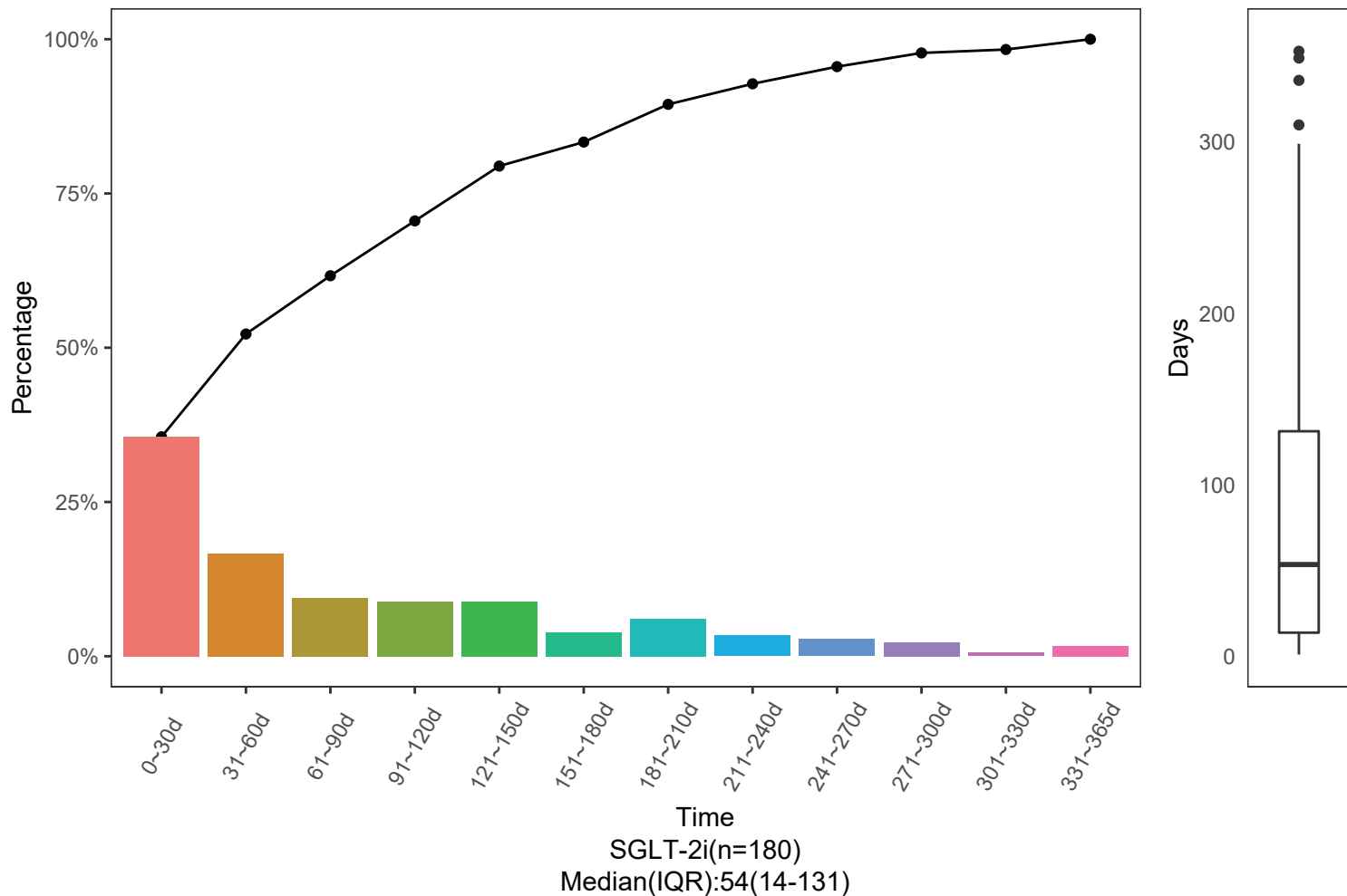

Supplementary Figure S1. Time interval between drug initiation and acute pancreatitis events.

Supplement: Supplementary file 2 [file Image1.pdf]
